# Surrogate sensitivity analysis of facet optical coatings produced without and with in situ design reoptimization

**DOI:** 10.1038/s41598-025-89625-6

**Published:** 2025-02-17

**Authors:** Daniel Poitras, Penghui Ma

**Affiliations:** Quantum and Nanotechnologies Research Center, National Research Council of Canada, 1200 Montreal Rd., Ottawa, ON K1A 0R6 Canada

**Keywords:** Robustness, Polynomial chaos expansion, Sobol indices, Optical coatings, Facet coatings, Applied optics, Engineering, Theory and computation

## Abstract

Optoelectronic and photonic waveguide-based devices often require the control of the exit/entrance facet reflectance to a high degree of precision on a relatively large wavelength range. Fabricating facet optical coatings for that purpose can be challenging due to thickness errors. In this work, a surrogate approach, the polynomial chaos expansion method, is used to evaluate the robustness of optical coating designs to experimental errors, and the Sobol’ sensitivity indices of their individual layers. The effect of a fabrication strategy involving successive in-situ design re-optimizations after completion of each individual layer deposition is simulated and shown to reduce significantly the detrimental effect of thickness errors on the yield and performance of coatings.

## Introduction

The sensitivity of some optical coatings to small accumulated thickness errors can complicate their fabrication and affect their performance. It is the case for example with large band, low reflectance coatings deposited on the facets of waveguide opto-electronic devices, generally sensitive to errors, sometimes forcing the design of devices with tilted facets^1–3^.

The sensitivity of coating designs to fabrication errors can be evaluated with analytical gradient expressions in the case of small thickness errors. This approach is local and neglect the combined effects of multiple layers thickness errors on the performance^4^. More generally, a Monte Carlo approach involving performance evaluations over a sampling of designs with errors can be used, but getting an accurate statistical estimation of the robustness and layer sensitivity requires a large sampling^5^. In addition to allowing the use of larger random thickness errors, the Monte Carlo approach also allows the use of complex simulation models with no simple analytical expressions, such as facet coatings reflectance simulation, or the introduction of more complex random events such as thorough coating deposition models taking into account fluctuations of deposition rates and optical monitoring signal errors, or in-situ design changes via re-optimization.

In this work, the robustness of some facet coating designs was evaluated using a surrogate approach, the polynomial chaos expansion (PCE) method^6^, in order to reduce the number of model evaluations and overall computation cost, while maintaining reliable robustness estimations^7^. A quantification of the performance sensitivity to each layer thickness was also performed. More importantly, the effect of in-situ design re-optimization during simulated fabrications of the coatings was studied. Part of this work was presented at the 2019 Optical Interference Coating Conference in Albuquerque NM^8^.

## Experimental methodology

### Semiconductor waveguide modal reflectance calculation

**Figure 1:**
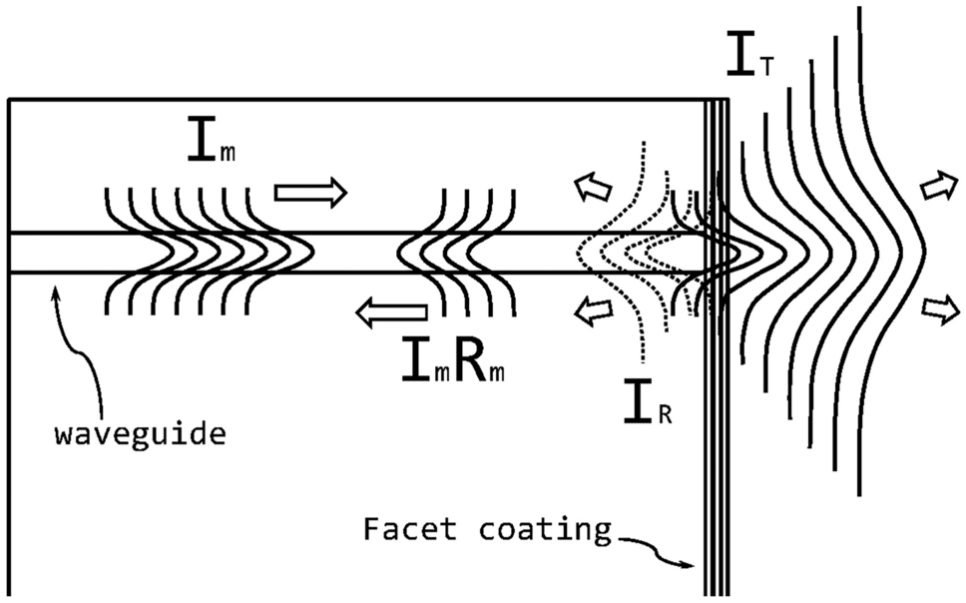
Schematic side view of a guided light mode exiting a waveguide and reaching a coated facet. An incident mode $I_m$ is decomposed into a distribution of planewaves that are either transmitted $I_T$ or reflected $I_R$ by the coating. The portion of the reflected light $I_R$ that is re-coupled into the waveguide forms the reflected mode $I_m R_m$.

The estimation of internal reflectivity of a waveguide mode incident on a facet requires more calculation than a straightforward normal incidence planewave problem^9^. Figure 1 schematize how one can approach this problem. It is important to understand that the waveguide mode entering the facet coating is no more guided; using the effective index of the mode as a substrate index or normal incidence only planewave calculations can lead to large performance evaluation errors.

Several methods have been developed for calculating the modal reflectance $R_m$ of waveguides. In this work, for designing and simulating the modal reflectance of a coated waveguide facet, we used a Python package called *CAMFR*, developed at Ghent University^10^ and based on an eigenmode expansion technique. For the demonstration purpose of this work, we used a simple InP-based waveguide structure, with a 250-nm core of InAs and semi-infinite InP claddings. The facet coatings were designed using $\hbox {SiO}_2$ and $\hbox {TiO}_2$ as coating materials.

### Simulation of coating fabrication, with and without in-situ design re-optimization

Simulation of fabrication errors was done by picking thickness errors $\Delta x_i$ on individual layer *i*, using normal distributions of errors with a standard deviation $\sigma$ around zero, and a quasi-random sampling sequence covering the space of possible performance alterations within normal distributions^11^.

**Figure 2:**
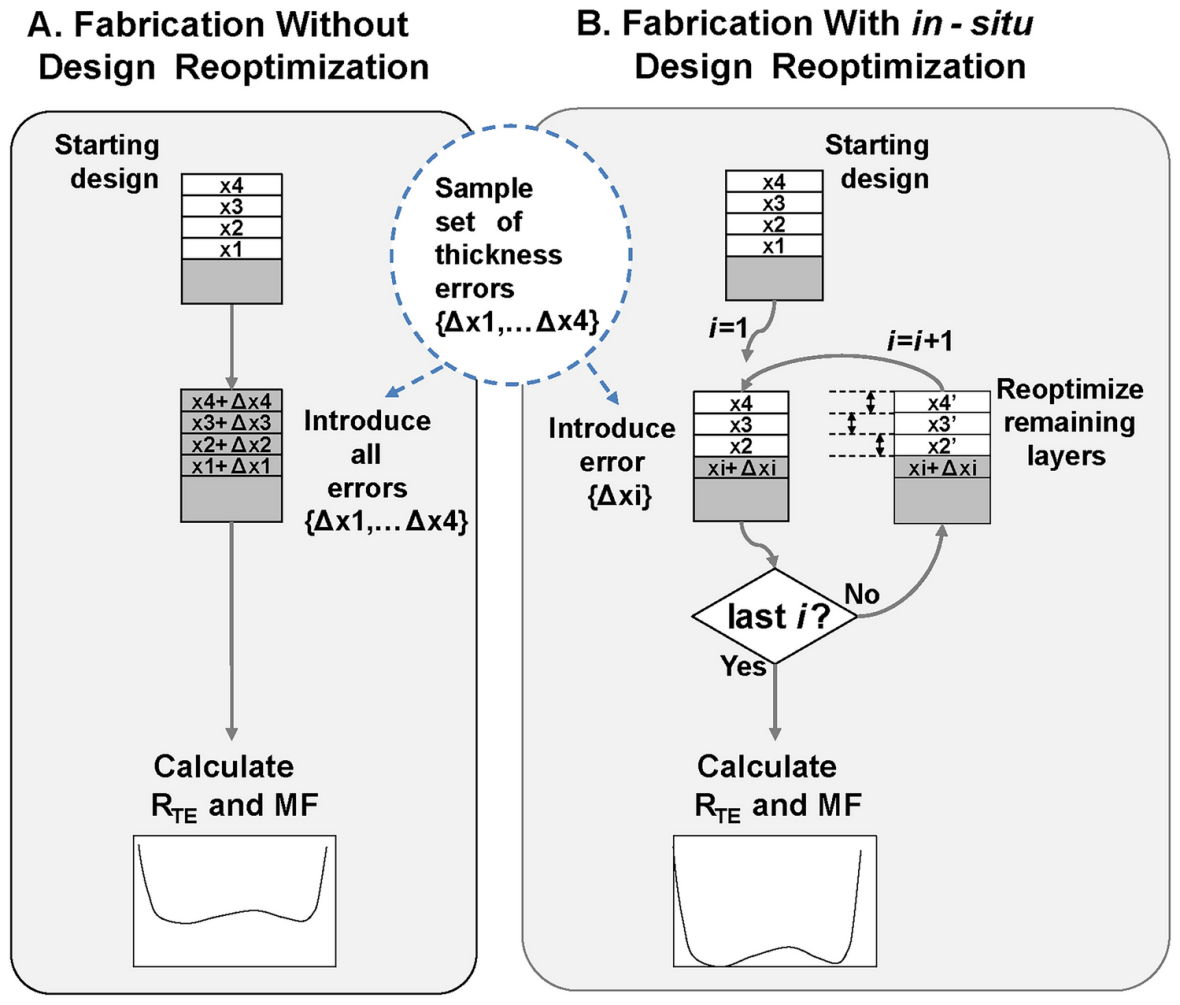
Steps for simulating the effect of fabrication errors on a 4-layer coating (**A**) without and (**B**) with the use of design re-optimization. One such cycle of simulation represents a sample in the Monte Carlo calculations.

Using this method, a sampling of multilayer systems with thickness errors was constructed and each set of errors was introduced to a design following two different schemes, as shown in Fig. 2. One scheme was intended to simulate fabrication of the coatings without in-situ design re-optimization (case A in Fig. 2); the thickness errors were applied simultaneously to all the layers in the design. In another scheme, each thickness errors were applied sequentially, and design re-optimization of the remaining layer thicknesses was done before introducing the next thickness error (case B in Fig. 2).

In situ design re-optimization, sometimes referred to as re-engineering, was proposed and implemented more than 30 years ago to mitigate the effect of accumulated layer thickness errors on the performance of fabricated coatings^12,13^. In machine learning terminology, in-situ re-optimization could be interpreted as a case of cyclic Reinforcement Learning^14,15^, where a design process is re-evaluated (re-optimized) when new information is known (here, exact thicknesses of deposited layers). In this work, we used a damped least-square method to re-optimize the design between fabrication steps.

### Polynomial chaos expansion and robustness evaluation

As mentioned in the introduction, a general Monte Carlo approach is required for evaluating the robustness of facet coating designs. Good accuracy, particularly when evaluating layers sensitivities, requires a large sampling of designs with errors, which translates into a large number of fabrication and performance simulations that are long to compute^5^, more so when in-situ design re-optimization is taken into account.

**Figure 3:**
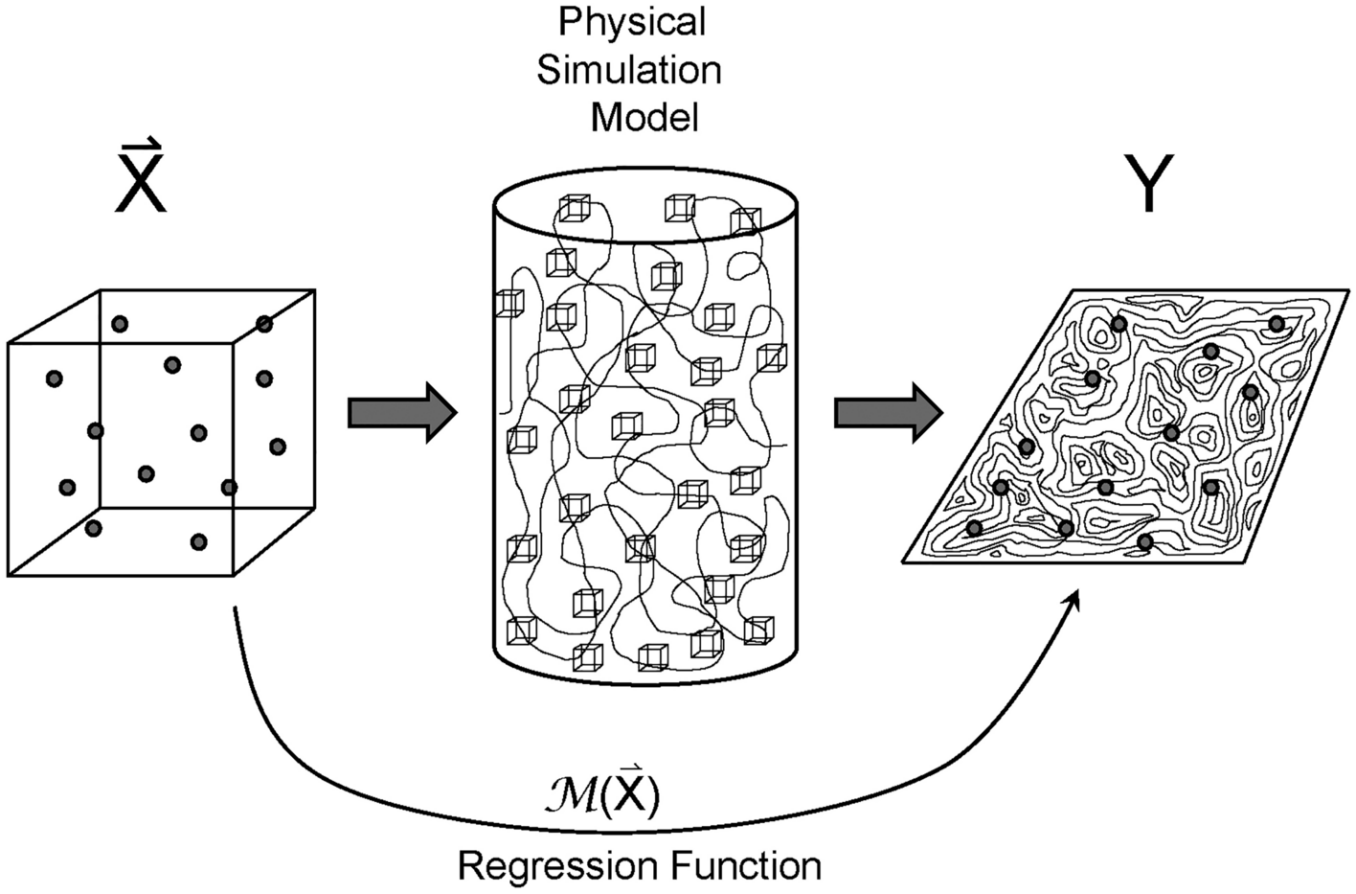
Schematic representation of the use of a surrogate model ${\mathcal {M}}(\vec {X})$ instead of complex physical model.

One approach for reducing the computation time consists in using a surrogate model, obtained by applying a regression on a reduced number of thickness-error samples $\vec {X} = \{\Delta x_1, \Delta x_2, \dotsc , \Delta x_M\}$ and their corresponding simulation results *Y* (Fig. 3). The less computer intensive surrogate model is then used in lieu of the simulation model to evaluate new samples or extract statistical properties. Several surrogate methods exist; in this work, the polynomial chaos expansion (PCE) spectral approach is used as a surrogate model for getting an accurate evaluation of the robustness while reducing the computation time^6,7^. That method is based on the following polynomial regression equation:

1 $$\begin{aligned} Y = {\mathcal {M}}(\vec {X}) = \sum _{\alpha \in {\mathbb {N}}^M} y_{\alpha } {\Psi }_{\alpha }(\vec {X}) , \end{aligned}$$

where

$$\begin{aligned} {\Psi }_{\alpha }(\vec {X}) \equiv \prod _{i=1}^{M} P_{\alpha _i}^{(i)}(x_i) \end{aligned}$$

are basis orthonormal functions consisting of multivariate polynomial equations with *P* being univariate Hermite polynomials (assuming gaussian distributions of errors $\Delta x_i$ for the parameters $x_i$), and $y_{\alpha }$ are coefficients to be computed with a regression, $\alpha = \{ \alpha _1, \alpha _2, \dotsc , \alpha _M \}$ being a multi-indices vector of natural numbers that has the dimension *M* corresponding to the number of model variables (number of layers), and giving the total degree of the polynomial as $p \le \sum _{i=1}^{M}\alpha _i$. The coefficients $y_{\alpha }$ and Eq. (1) can be used to evaluate *Y* data from additional error vectors $\vec {X}$. A few software applications are available for PCE calculations. We used *Chaospy*, a python package developed at University of Oslo^16^; UQ[py]lab is another possible option, using cloud computing^17^ at ETH Zürick.

To evaluate the performance of each calculated spectrum $R_\textrm{calc}(\vec {X}, \lambda )$, we used a Merit Function (*MF*), defined as:

2 $$\begin{aligned} { MF }(\vec {X}) = \frac{1}{N_\lambda } \left\{ \sum _{\lambda _i} \left[ \dfrac{R_\textrm{calc}(\vec {X}, \lambda _i)-R_\textrm{target}(\lambda _i)}{\Delta R(\lambda _i)} \right] ^2 \right\} ^{1/2} \end{aligned}$$

where $\lambda _i$ represents the wavelengths defining the target curve $R_{\textrm{target}}$, and $N_{\lambda }$ is the number of wavelengths. A lower *MF* value corresponds to a better performance. To visualize and compare the robustness of different designs and approaches, we chose to show (i) a compilation of calculated spectra, and (ii) cumulative histogram showing fraction of the spectra having a performance better than a certain *MF* value [an estimation of the ’probability of success’ (PoS)].

### Sobol’ sensitivity indices

In addition to evaluating the robustness of a design, we used the sampling $\vec {X}$ and data $Y= MF (\vec {X})$ to perform a sensitivity analysis, using Sobol’ sensitivity values^18,19^:

3 $$\begin{aligned} S_{\alpha } \overset{{\textrm{def}} }{=} \frac{Var[{\mathcal {M}}_{\alpha }(\vec {X}_{\alpha })]}{D}, \end{aligned}$$

which represent variances of the performance on the parameter vectors $\alpha$ when all other parameters are kept constants, normalized by the variance $D = \sum _{\alpha }S_{\alpha }$ of the performance when all parameters are varied. In this work, we used the first order Sobol’ indices $S_i$ that describe the variance of *Y* when only the variation of layer *i* is considered, and also the total Sobol’ sensitivity indices, defined as:

4 $$\begin{aligned} S_i^{t} \overset{{\textrm{def}} }{=} \sum _{\alpha \supseteq i}S_{\alpha } = 1 - \sum _{\alpha \nsupseteq i}S_{\alpha }, \end{aligned}$$

which represent the sum of all the variance and covariance values involving the layer *i*. We also used the second order Sobol’ sensitivity indices $S_{i,j}^{t}$ (with $i\ne j$), defined as:

5 $$\begin{aligned} S_{i,j}^{t} \overset{{\textrm{def}} }{=} \sum _{\alpha \supseteq i,j}S_{\alpha }-S_{i}-S_{j}, \end{aligned}$$

which represent the sum of the covariance values simultaneously involving the layers *i* and *j*.

One can compute Sobol’ indices stricly from a large Monte Carlo sampling of model evaluations^20^. However, this method is computer intensive, and thanks to similarities between the regression model ${\mathcal {M}}$ and the Sobol’ decomposition, the PCE approach allows a direct determination of the Sobol’ sensitivity indices from the coefficients $y_\alpha$ in Eq. (1)^21^. This reduces drastically the computational time required for the sensitivity analysis and is the main reason for using the PCE approach in this study.

## Results and discussion

**Figure 4:**
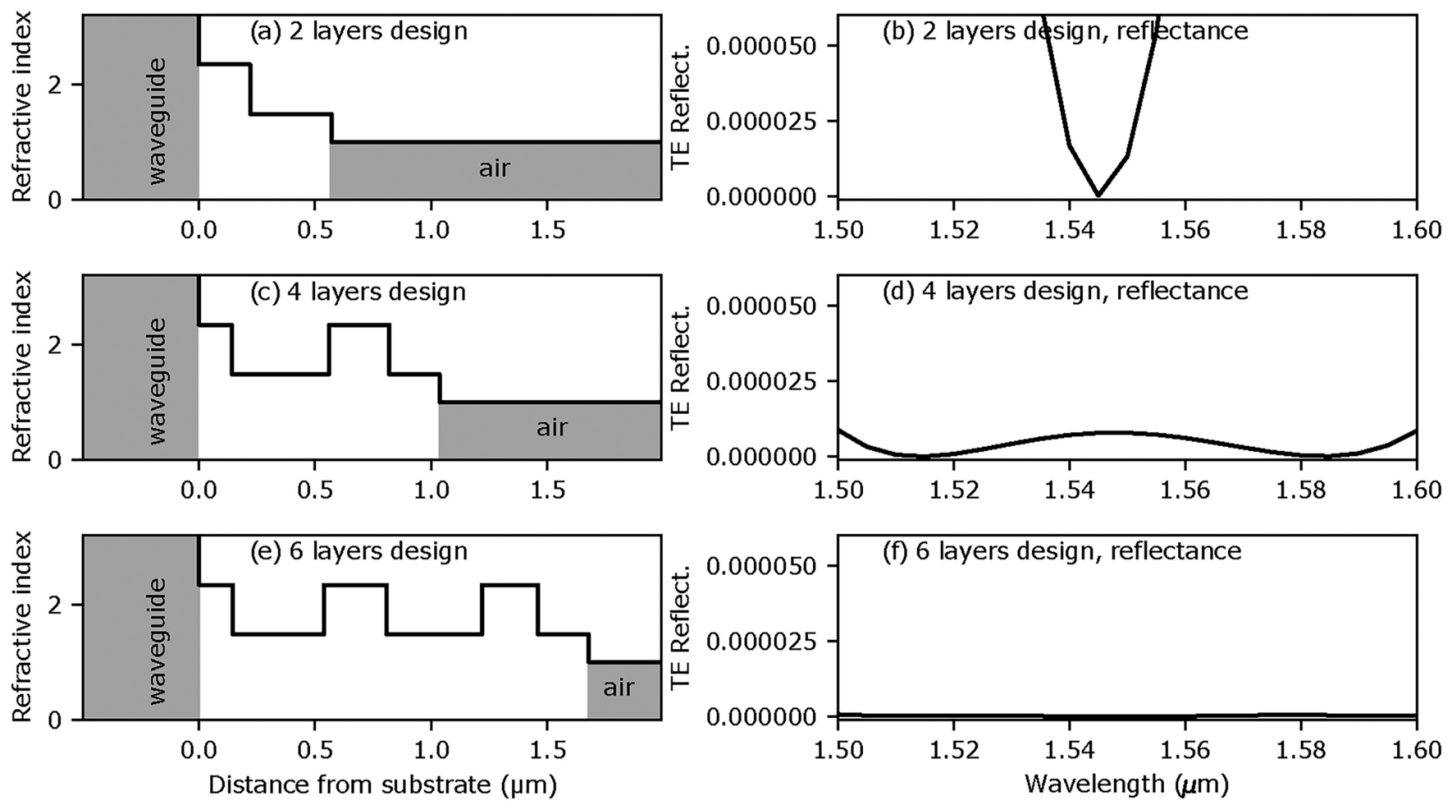
Facet AR coating designs and corresponding TE modal reflectance (ideal, no thickness errors): (**a,b**) 2-layer ($WG|221.6nm\, H|348.8nm\, L|air$), (**c,d**) 4-layer ($WG|141.5nm\,H|419.3nm\, L|257.7nm\,H|216.4nm\, L|air$) and (**e,f**) 6-layer ($WG|147.0nm\, H|391.7nm\, L|270.0nm\, H|409.2nm\, L|240.4nm\, H|218.7nm\, L|air$) designs. WG represent the waveguide, *H* is $\hbox {TiO}_2$ and *L* is $\hbox {SiO}_2$.

Figure 4 shows AR facet coating designs with of 2, 4 and 6 layers, and their respective expected performance, without thickness errors. The 2-, 4- and 6-layer designs were obtained from optimizing the thickness values of manually set starting designs (minimizing Eq. 2), first coarsely with a damped least-square method and then finely with a Hooke and Jeeves pattern search method. This design process was repeated a few times with new starting designs in order to find stable solutions (isolated local minima of Eq. 2). We used the 2- and 6-layer designs to demonstrate the robustness and sensitivity analysis approach and the effect of in-situ design re-optimization.

In practice, choosing between 2- and 6-layer designs depends on the required performance. In this work, as described in the next Sections, we assumed different reflectance specifications for the 2- and 6-layer designs. In both cases, evaluating the performance of the coating was done using Eq. (2) with a zero-reflectance target and a constant tolerance of $\Delta R = 1\times 10^{-5}$, for wavelengths between 1.5 $\upmu$m to 1.6 $\upmu$m.

**Figure 5:**
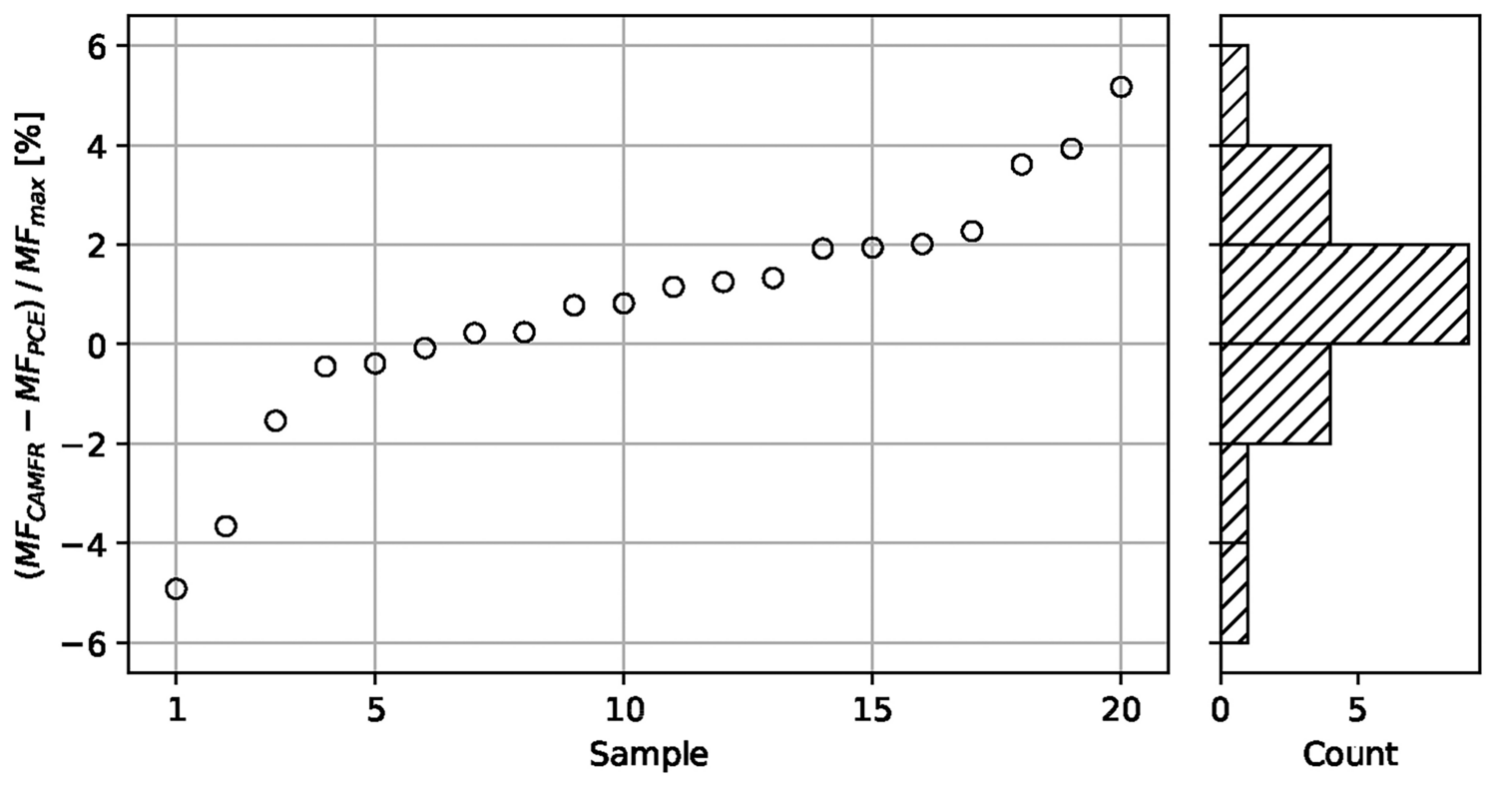
Validation of the PCE model for a 6-layer AR design: difference of the *MF* values from the PCE and CAMFR models, relative to the maximum acceptable *MF* for that design.

We applied the PCE approach and sensitivity analysis to the different facet antireflective (AR) coating designs. In all cases, thickness errors were selected quasi-randomly using a Hammersley (*H*) sequence^22^, assuming Gaussian distributions of thickness errors centered at zero, with the following standard deviations $\sigma$: (i) 1-nm for layers with thickness $d_i$ > 100 nm, and (ii) 1%$\times d_i$ for layers with thickness $d_i$ $\le$ 100 nm. This operation was repeated for a sampling of $N = 100$ designs with errors. Then, simulations of the reflectance and *MF* value evaluations were done for each of these designs, using the methods described above (Fig. 2). Finally, a PCE model was calculated, validated, and used to extract statistical metrics. The validation consisted in calculating the difference of *MF* values from the PCE model and the more rigorous CAMFR model, for data points outside of the sampling set used for defining the PCE model, and measuring its impact on the robustness evaluation by comparing this difference to the maximum acceptable *MF* value for this design ($MF _\textrm{max}$). Figure 5 shows the validation for the 6-layer design (for which $MF _\textrm{max}=5$, see “Case 2: 6-layer AR design”), performed on 20 samples; we see that using the PCE model has a relatively small impact on the performance evaluation, with absolute errors on *MF* limited to less than 6% of $MF _\textrm{max}$ value.

### Case 1: 2-layer AR design

The design is described on Fig. 4a and its caption. As a specification, we assumed that the acceptable reflectance had to be lower than $1\times 10^{-3}$ from 1.5 $\mu \hbox {m}$ to 1.6 $\mu \hbox {m}$, corresponding to *MF* upper limit $MF _\textrm{max} = 100$. The merit function of the design (i.e. error-free coating) was $MF = 70.1$.

**Figure 6:**
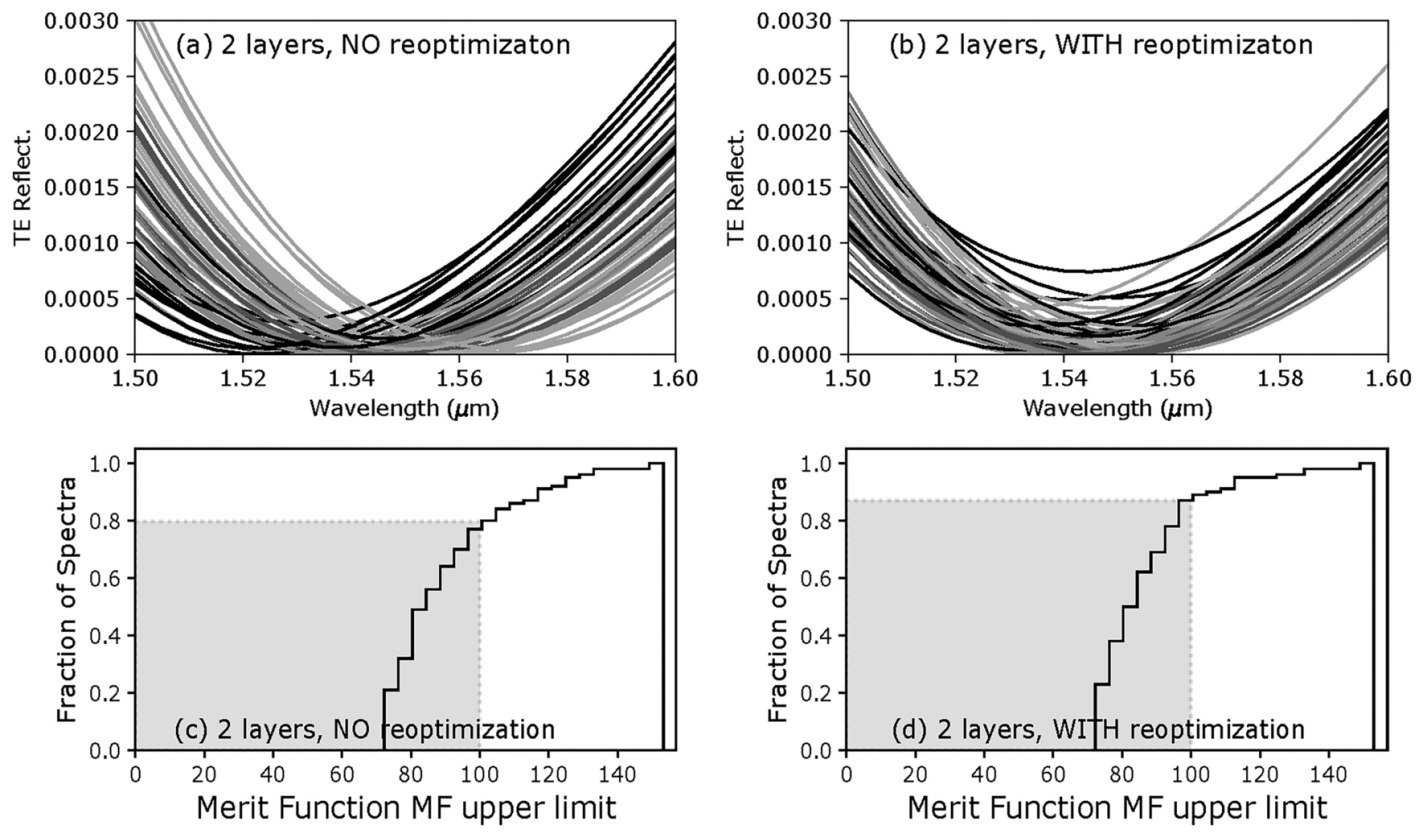
Calculated TE reflectance for all 2-layer design-samples, and corresponding cumulative histograms, for fabrication simulations without and with re-optimization.

The reflectance spectra calculated from the same initial layer errors sampling are shown in Fig. 6a, b for cases without and with in-situ design re-optimization. The effect of re-optimizing the design during the fabrication is small, but obvious: the reflectance spectra are shifted back towards the central wavelength, keeping the overall reflectance lower within the wavelength range specified. In terms of performance, the cumulative *MF* histograms are shown in Fig. 6c, d, and due to the shifts of spectra and their rectification when using re-optimization, the *PoS* goes from 80% to 85% in this case. Clearly, given the wavelength range and the required $1\times 10^{-3}$ reflectance, 2 layers in a design can hardly show the benefice of in-situ design re-optimization; once the first layer is deposited with error, only the last layer can be adjusted to rectify the spectrum.

### Case 2: 6-layer AR design

Figure 4c shows a 6-layer design. Assuming that reflectance lower than $5\times 10^{-5}$ is targeted between 1.5 $\upmu$m and 1.6 $\upmu$ gives an upper limit of $MF _\textrm{max}=5$ for acceptable coating performance (*MF* of the design without errors was 0.0365).

**Figure 7:**
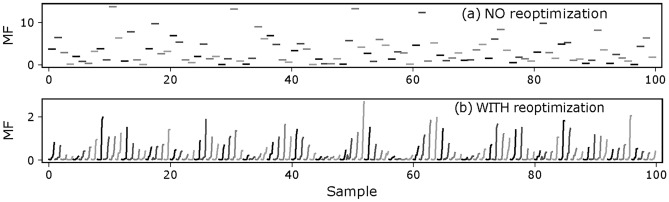
Evolution of *MF* for the 6-layer design samples.

Figure 7 compares the *MF* values for 100 design samples with errors, simulated without and with design re-optimization (Fig. 2). In the cases without re-optimization, each sample has a single *MF* value obtained after applying in parallel (simultaneously) all the thickness errors to the layers. In the cases using re-optimization, for each sample, the thickness errors are introduced in series and design re-optimization reduced their effect on *MF*, resulting in a slow increase of *MF* with the addition of layers, and a final *MF* lower than the case without re-optimization (using identical error samples). In addition, one can see the quasi periodicity of the *MF* distribution amongst the design error samples; this is due to the fact that we are not using a purely random sampling but rather a quasi-random Hammersley (*H*) sequence (it has generally a better coverage of the parameter space).

**Figure 8:**
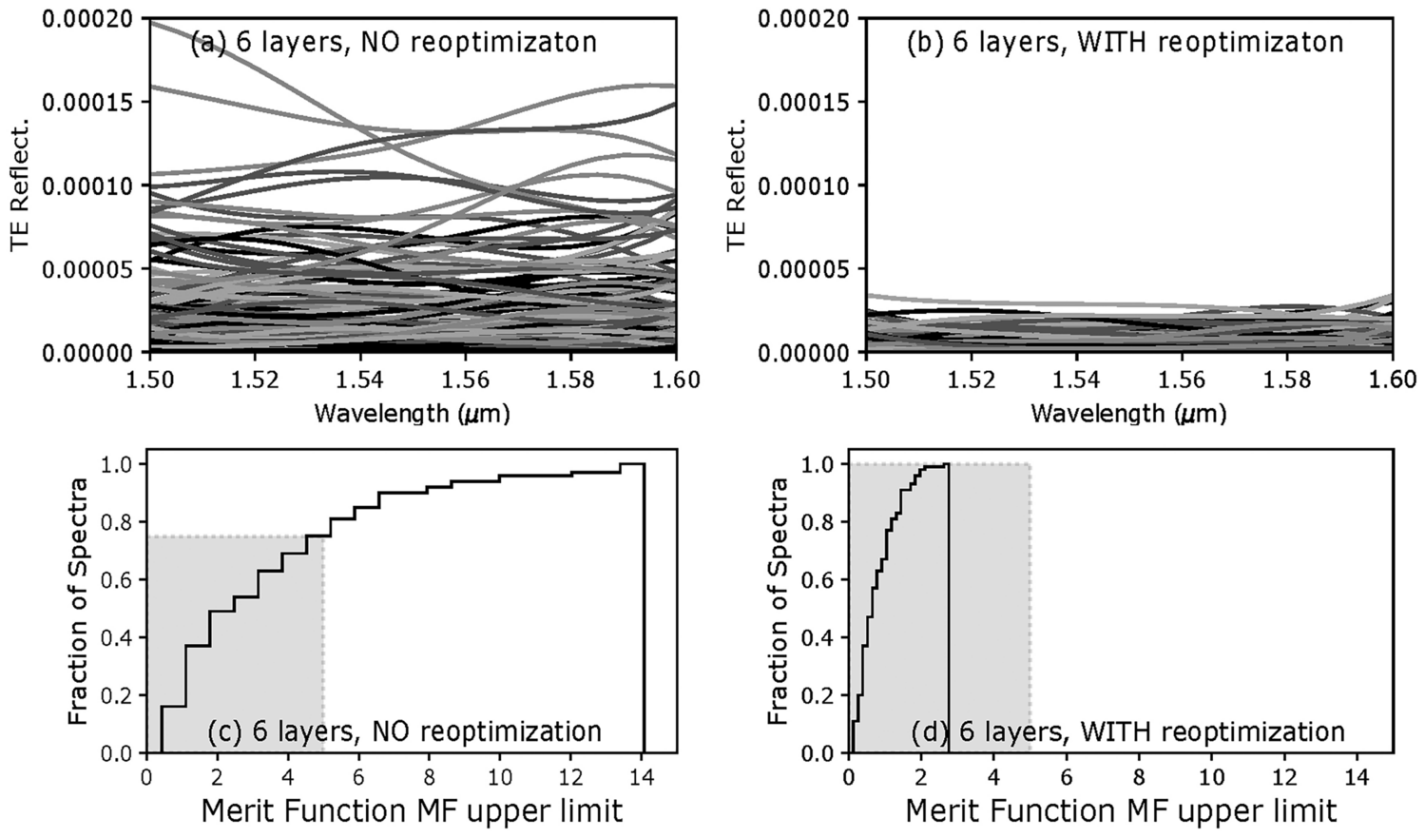
Calculated TE reflectance and cumulative histograms for all 6-layer design-samples, without and with re-optimization.

Similarly to Fig. 6, Fig. 8a, b show the reflectance spectra corresponding to each error sample. In the case of the 6-layer AR design, the reflectance spectra of all the samples are lower than $4\times 10^{-5}$ when re-optimization is used, a maximum value 5 times lower than that of spectra calculated without design re-optimization (both with identical layer thickness errors!) It shows that the more layers a design has, the more degrees of freedom there is to correct for thickness errors in its first layers when using design re-optimization.

The resulting *MF* values, without and with re-optimization, are shown on the cumulative histograms of Fig. 8c, d. We can see that using re-optimization during fabrication can increase the *PoS* from 75% to 100% in that 6-layer design case.

**Figure 9:**
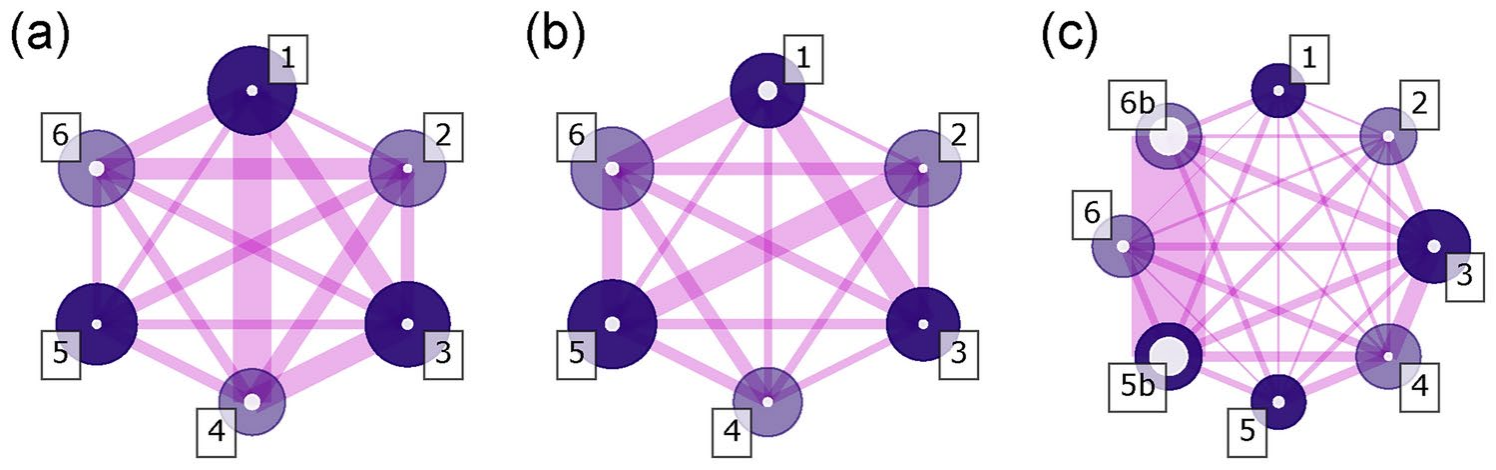
Network diagrams showing *MF* sensitivity interdependence of a 6-layer design, (**a**) without re-optimization, (**b**) with re-optimization, and (**c**) with re-optimization and layer subdivisions (last 2 layers). The nodes represent the layers, starting with the layer next to the substrate (darker nodes represent *H* layers). The diameter of large colored and small white circles at the nodes are proportional to ${ S}^{t}_i$ and ${ S}_i$ indices, respectively, and the thickness of the segment joining layers *i* and *j* is proportional to ${ S}_{i,j}$ indices.

As mentioned previously, Sobol’ sensitivity indices help in finding the layers with the most impact on the performance. Using different PCE models, one could evaluate Sobol’ coefficients either as a function of wavelength and see the effect of layers thicknesses on different regions of the spectrum, or directly on the *MF* value, the approach taken here. PCE models for the 6-layer cases were used to extract Sobol’ indices (Eqs. 3, 4 and 5). Figure 9 shows network diagrams where nodes and segments represent Sobol’ indices of degree 1 and 2, respectively (see figure caption for details). We see on Fig. 9 that when fabricating this coating without re-optimization, the most critical layers are layers 1 and 3 (larger node radii), and the most important combined effect is from layers 1 and 4. Figure 9b shows the effect of introducing design re-optimization on the network diagram; as expected, it pushes the sensitivity towards the last layers 5 and 6. It should be noted that, since the sensitivity parameters are both normalized to the total variances values for each models (Eq. 3), the Sobol’ indices cannot be directly compared between models (i.e. nodes diameters in Fig. 9a, b). Table 1 compiles the variance values extracted from the 6-layer design simulations; multiplying the Sobol’ indices (nodes diameter and segments width) by the respective total variance *D* allows a comparison of the sensitivities between models.

**Figure 10:**
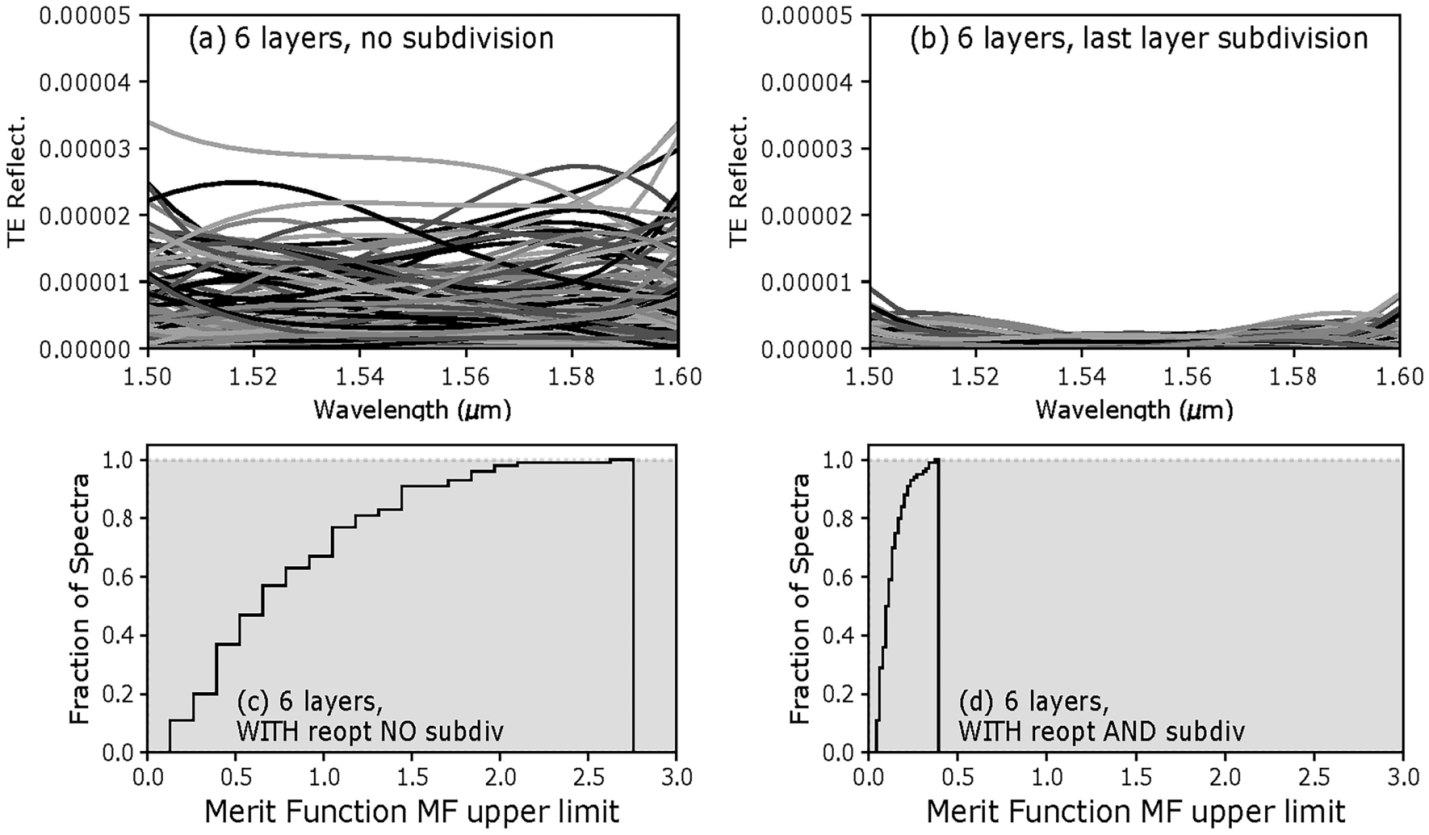
Effect of subdividing the last two layers of a 6-layer design on its MF cumulative histogram: (**a**) with re-optimization and no subdivision, (**b**) with re-optimization and subdivision of the last 2 layers (both subdivided in 2 sublayers, and ending with 15 nm-thick sublayers).

One way to reduce the thickness error on sensitive layers when using re-optimization is to subdivide them into sublayers, ending with a thin sublayer. This subdivision makes the thinnest last sublayer the critical layer, with a thickness error that is less than that of the not-divided thicker layer. To demonstrate this effect, we subdivided by two the last two layers of the 6-layer design, ending both layers with 15-nm sublayers (with thickness $\sigma$ of 0.15 nm). We used the same PCE and Sobol’ indices approach as above on this new 8-layer system. The reflectance spectra and cumulative histogram are shown in Fig. 10b, d, revealing the beneficial effect of the strategy for reducing the thickness errors on the reflectance spectra and *MF* values.

Figure 9c and Table 1 show the effect of design re-optimization and layer subdivision on the Sobol’ indices and the variance of *MF*. The table shows that the overall sensitivity was reduced significantly, and the graph shows that the remaining sensitivity was been mainly transferred to the 15-nm sublayers (first and second order Sobol’ indices).

**Table 1:** Values of the Sobol’ indices multiplied by the total variance *D* for the 6-layer design, and fabrication simulation model without and with design re-optimization, and with layer subdivisions.

Model	Lay.*i*	${D\times S}_i$	${D\times S}^{tot}_i$	${D\times S}^{tot}_{i,j}$						
				(*i*, 2)	(*i*, 3)	(*i*, 4)	(*i*, 5)	(*i*, 5*b*)	(*i*, 6)	(*i*, 6*b*)
NO REOPT ($D = 3.16$)	1	0.0204	1.99	0.0185	0.0705	0.1230	0.0244		0.0490	
	2	0.0131	1.50		0.0417	0.0588	0.0461		0.0692	
	3	0.0232	1.85			0.0793	0.0316		0.0380	
	4	0.0537	1.14				0.0379		0.0354	
	5	0.0145	1.68						0.0278	
	6	0.0465	1.50							
REOPT ($D = 0.148$)	1	0.00355	0.0657	0.000639	0.00425	0.00125	0.00104		0.00361	
	2	0.000565	0.0707		0.00172	0.00135	0.00426		0.00191	
	3	0.000712	0.0639			0.00120	0.00157		0.00178	
	4	0.000780	0.0571				0.00169		0.00201	
	5	0.00201	0.0940						0.00300	
	6	0.00172	0.0826							
REOPT & SUBDIV ($D =0.00198$)	1	1.12E−05	4.57E−04	5.07E−06	1.08E−05	7.50E−06	3.82E−06	1.17E−05	2.18E−06	1.01E−05
	2	1.32E−05	5.17E−04		1.85E−05	7.62E−06	4.83E−06	1.24E−05	5.82E−06	7.90E−06
	3	1.87E−05	8.55E−04			3.50E−05	1.04E−05	1.82E−05	1.67E−05	1.76E−05
	4	7.48E−06	6.79E−04				1.91E−05	2.06E−05	1.59E−05	6.63E−06
	5	1.65E−05	4.71E−04					1.36E−05	3.84E−06	1.19E−05
	5b	2.24E−04	7.15E−04						1.71E−05	1.49E−04
	6	1.70E−05	6.20E−04							1.53E−05
	6b	2.14E−04	6.93E−04							

## Conclusion

In this paper, we used of a surrogate method, polynomial-chaos expansion, for the evaluation of optical coating designs robustness and individual layer thickness errors sensitivity, in the case of single-mode waveguide facet large-band AR coatings. The PCE method particularly simplify the calculation of Sobol’ indices giving an estimate of the coating’s performance dependence and co-dependence on individual layer thickness errors.

We used the surrogate method to demonstrate that a fabrication approach including design re-optimization can have a significant impact on the expected performance of even few-layer optical coatings, improving favorably the Sobol’ indices related to layers thickness errors. Introducing design re-optimization to the fabrication simulation makes the model dynamic, with a design that changes during a simulation; it was interesting to note that the surrogate model still works well in that case.

Clearly, surrogate methods could be applied to other types of optical coatings, i.e. (i) optical coatings with a large number of layers, for which thorough sensitivity analysis can be computationally costly, or (ii) coating controlling the phase dispersion, particularly sensitive to thickness errors. A special attention may be required for visualizing the overall layer sensitivities for multilayers with a large number of layers.

## Data Availability

The datasets generated during and/or analyzed during the current study are available from the corresponding author on reasonable request.
